# Supplementary material for: Reactivation of Hepatitis B Virus in Lung Cancer Patients Receiving Tyrosine Kinase Inhibitor Treatment
Source: J Clin Med. 2022 Dec 28;12(1):231. doi: 10.3390/jcm12010231 (PMC9820864; doi:10.3390/jcm12010231)
Supplement: Supplementary file 1 [file jcm-12-00231-s001.zip › jcm-2031160-supplementary.pdf]

	Reactivation	No reactivation	P value[a]
	(n = 16)	(n = 333)	
**Age, years, median (IQR)**	59.5 (53.5 – 67.0)	67.0 (58.0 – 76.0)	0.014
**Gender, n (%)**			0.605
Male	8 (50%)	137 (41.1%)	
Female	8 (50%)	196 (58.9%)	
**Smoking, n (%)**			0.171
Never	10 (62.5%)	234 (70.3%)	
Ever	6 (37.5%)	69 (20.7%)	
Not available	0 (0.0%)	30 (9.0%)	
**Histology, n (%)**			1.000
Adenocarcinoma	16 (100%)	320 (96.0%)	
Others	0 (0.0%)	13 (3.9%)	
**Tyrosine kinase inhibitor, n (%)**			0.775
Gefitinib	6 (37.5%)	79 (23.7%)	
Erlotinib	6 (37.5%)	135 (40.5%)	
Afatinib	3 (18.8%)	74 (22.2%)	
Osimertinib	1 (6.3%)	22 (6.6%)	
ALK-inhibitor	0 (0.0%)	23 (6.9%)	
**HBV reactivation follow up duration (months), median (IQR)**	11.2 (3.3 – 22.0)	15.4 (7.0 – 27.9)	0.177
**HBV serology, n (%)**			<0.001
Negative HBsAg	1 (6.3%)	275 (82.6%)	
Positive HBsAg	15 (93.8%)	58 (17.4%)	

Supplementary Table S1 Characteristics of lung cancer patients receiving tyrosine kinase inhibitor treatment only with or without HBV reactivation. [a]Fisher's exact test, Pearson’s chi-square test, and Mann–Whitney U test

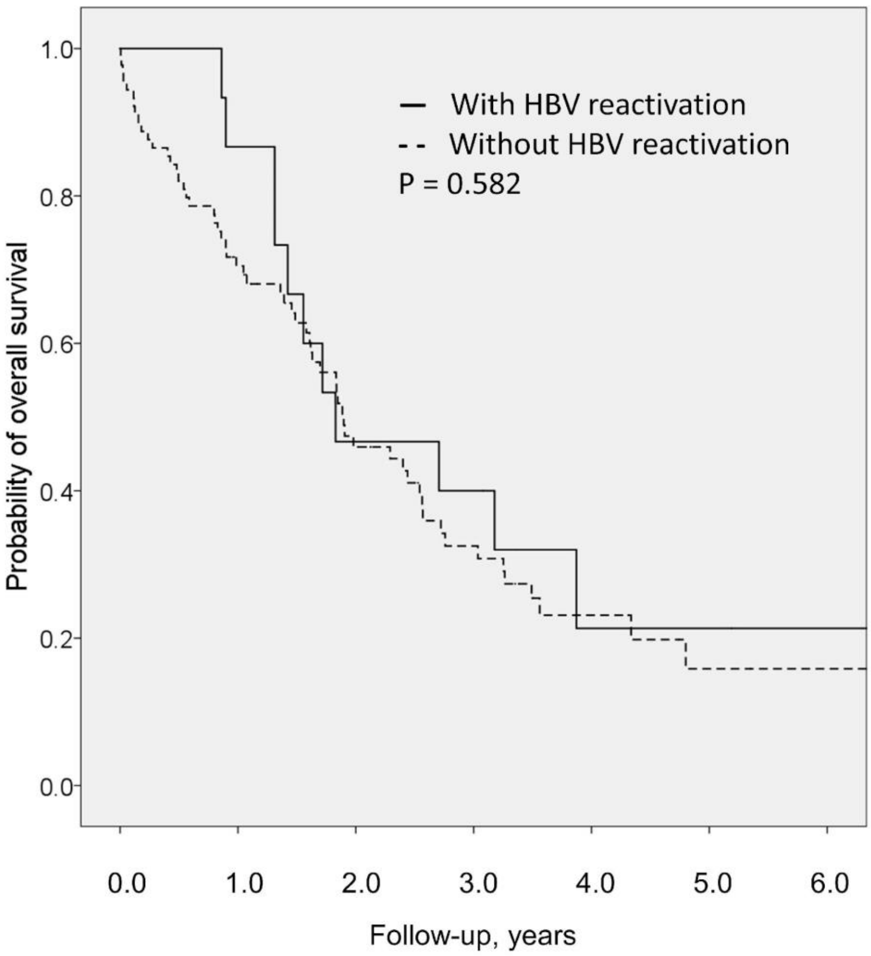

No. at risk							
With HBV reactivation	15	13	7	6	2	2	1
Without HBV reactivation	89	59	31	19	9	4	3

Supplementary Figure S1 Overall survival of HBsAg-positive lung cancer patients receiving first-line tyrosine kinase inhibitor treatment with and without HBV reactivation
